# Distribution of Methylene Blue after Injection into the Epidural Space of Anaesthetized Pregnant and Non-Pregnant Sheep

**DOI:** 10.1371/journal.pone.0092860

**Published:** 2014-04-07

**Authors:** Xavier Moll, Felix García, Rosa Isabel Ferrer, Laura Santos, Adrià Aguilar, Anna Andaluz

**Affiliations:** Departament de Medicina i Cirurgia Animals, Facultat de Veterinària, Universitat Autònoma de Barcelona, Bellaterra, Spain; Xavier Bichat Medical School, INSERM-CNRS - Université Paris Diderot, France

## Abstract

The aim of the study was to determine the distribution of different volumes of methylene blue solution injected into the epidural space in anaesthetized pregnant and non-pregnant sheep, to evaluate its cranial distribution and to compare between them. Fifteen pregnant and fifteen non-pregnant sheep were included in the study. Sheep were anaesthetized and received 0.05, 0.1, or 0.2 mL/kg of a lumbosacral epidural solution containing 0.12% methylene blue in 0.9% saline. Thirty minutes after the epidural injection, the ewes were euthanized. The extension of the dye within the epidural space was measured, and the correlation between the volume of the dye injected and the number of stained vertebrae was evaluated. The cranial migration of the dye between pregnant and non-pregnant sheep was also compared. The results show that the volume of methylene blue injected epidurally into pregnant and non-pregnant sheep correlated directly with its cephalic distribution into the epidural space; and a volume of 0.1 mL/kg or 0.2 mL/kg stained up to the first lumbar segment in pregnant and non-pregnant sheep, respectively. Also, the results suggest that the volume of drugs administered into the epidural space of pregnant sheep should be half the volume that would be used in non-pregnant sheep.

## Introduction

One of the most common regional anaesthetic techniques used to control pain in many species is lumbosacral epidural anaesthesia. This procedure involves the injection of an analgesic/anaesthetic solution into some part of the spinal canal to obtain regional analgesia/anaesthesia [1], [2]. Lumbosacral epidural anaesthesia is indicated for surgical procedures caudal to the umbilicus [1], such as caesarean section associated with vaginal and/or rectal prolapse or fetal malposition in pregnant sheep; and improves the surgical and postoperative conditions for caudal intra-abdominal, pelvic, or hind-limb surgery in pregnant and non-pregnant sheep. When epidural anaesthesia is used in association with general anaesthesia, it reduces the requirement for anaesthetics and promotes residual analgesia for up to 12–24 hours [3]–[5].

During epidural anaesthesia, the action of local anaesthetic drug is on the sodium channels expressed on spinal nerves. The site of action of local analgesics given epidurally is controversial, but the sensory and motor blockade is attributed to the direct contact with the superficial layers of the nerve roots in the spinal cord [6], [7]. The migration of drugs within the epidural space can be influenced by different factors, including the injection volume, rate of infusion, intervertebral foramina, body position, epidural fat, vascular absorption, and the anatomy of a particular species [8]–[10]. Over the last 10 years, the cranial spread of methylene blue (MB) into the epidural space has been studied in several species [9]–[13], and a correlation between the volume administered and the cranial distribution of the dye has been observed.

Finally, epidural anaesthesia is very useful in small animals and ruminants for obstetric surgeries, particularly caesarean sections. When such animals are pregnant, a 25–33% reduction in the volume of drug used in non-pregnant animals is recommended [1], [14]. However, to our knowledge, no studies have been performed to evaluate epidural migration of MB in pregnant animals. The aim of this study was to investigate the cranial distribution of 3 different volumes of MB, selected regarding a previous study in non-pregnant sheep [15], injected into the lumbosacral epidural space in pregnant and non-pregnant sheep. We hypothesized that there is a correlation between the volume of MB administered and the migration and cranial distribution of MB through the epidural space of pregnant ewes, similar to that observed previously in non-pregnant sheep. Although there are not studies that compare the migration of the drugs injected into the epidural space between pregnant and non-pregnant animals, we think that a reduction of a 25–33% of the volume of MB to dye the same vertebral space in pregnant ewes regarding non-pregnant sheep will be observed in our study.

## Materials and Methods

The prospective experimental trial was approved by the Ethical Commission of Animal and Human Experimentation (Spanish Government, Authorization Numbers DARP5332 and DARP6123) under the auspices of the Ethical Commission of the Autonomous University of Barcelona. Thirty 4- to 5-year-old Ripollesa sheep were enrolled in this study. Fifteen non-pregnant sheep weighing 44.67±4.79 kg (mean ± SD), and fifteen pregnant ewes carrying singleton fetuses weighing, ewe and fetus, 47.6±3.4 kg were included in the study. The mean gestational age of pregnant ewes was 121.4±1.75 days (range, 119–123 days; full term, 147–150 days). Non-pregnant sheep were destined for slaughter and were only selected when a diagnosed disease did not affect the spinal cord and/or epidural space. Pregnant ewes were the final part of another study, a pharmacodynamic and pharmacokinetic research project which evaluated the effect of an intravenous hypnotic agent in the cardiovascular system of the mother and fetus. These ewes were included in the study 24 hours after finishing the previous study. All animals were randomly assigned to 3 groups (n = 10 per group, 5 non-pregnant and 5 pregnant). MB (0.12%) in 0.9% saline was administered epidurally at the lumbosacral junction (L7–S1) as follows: group 1, 0.05 mL/kg; group 2, 0.1 mL/kg; and group 3, 0.2 mL/kg.

Non-pregnant sheep were deprived of food and water for 24 h prior to the anaesthesia. Pregnant sheep were also deprived of food and water for 24 h prior to the anaesthesia of our study. During the previous study, pregnant animals were not deprived of food and water, and received lactated Ringer's solution during 240 minutes (study period).

The anaesthesia was induced in all animals by administration of a single dose of 1 mg/kg etomidate intravenously through an 18-gauge polyurethane catheter placed in the cephalic vein. The animals were intubated with a 9–10 mm endotracheal tube, and anaesthesia maintained with 1.5–2% isoflurane in 100% oxygen using a semi-closed circular anaesthetic system. An orogastric tube was inserted while the sheep were anaesthetized to prevent rumen tympany. The animals were positioned in sternal recumbency with the hind limbs pulled slightly forward. The sheep remained in this position throughout the rest of the experiment and whilst the laminectomy was being performed. The skin on the lumbosacral space was aseptically prepared. Epidural injection was administered using a 20-gauge, 88-mm spinal needle (Spinocan, B.Braun, Rubí, Spain). The point of injection was located between the spinous process of the seventh lumbar vertebra and the sacrum. The needle was inserted perpendicular to the midline sagittal plane and with the needle bevel directed cranially. The correct needle placement was confirmed by the hanging-drop method, by the absence of cerebrospinal fluid or blood after aspiration, and by noting no resistance during MB solution injection [2]. All injections were performed by the same investigator, and the total time taken to administer the injection was 60–65 seconds.

All animals received an infusion of lactated Ringer's solution at a rate of 10 mL/kg/h during the period of anaesthesia. Heart rate, respiratory rate, pulseoximetry, capnography, and noninvasive blood pressure of the sheep (the cuff was placed at the proximal third of the radius to measure the pressure in the brachial artery), and heart rate and invasive blood pressure of the fetus were monitored during anaesthesia (by a catheter placed into the carotid artery in the previous study) using a multiparametric monitor (VetCare Multiparamétrico, B.Braun, Rubí, Spain).

Thirty minutes after the epidural injection, the animals were euthanized with pentobarbital sodium (100 mg/kg IV). The death of the sheep was confirmed by the lack of heartbeat, lack of electrical activity of the heart determined by ECG, and lack of respiration. The death of the fetus, in pregnant ewes, was confirmed by the absence of arterial pressure waveform and heart rate through a catheter inserted, during the previous study, into the carotid artery.

At necropsy, a dorsal laminectomy between the middle thoracic and the last sacral vertebra was performed. The dorsal arch of each vertebra was removed and the blood was collected with a suction system to prevent blood contamination of the epidural space. A second investigator, blinded to all treatments, measured the cranial and caudal migration of the dye within the epidural space from the injection site by examining the staining of the epidural fat and dura mater on both sides of the sagittal section, the distance (in cm) of the dye from the injection point was measured. The number of stained vertebral body segments was counted as in others studies [16], [17], by rounding the number to the nearest cranial intervertebral space to which the dye had migrated as L7 = 1, L6 = 2, L5 = 3, L4 = 4, L3 = 5, L2 = 6, L1 = 7, T13 = 8, T12 = 9, T11 = 10, T10 = 11, T9 = 12, T8 = 13, T7 = 14, and T6 = 15. If the spread was different between right and left side, the mean of the two spreads was taken in to account. The vertebra was not counted if less than half of it was stained.

### Statistical analysis

Data were analyzed using a statistical computer software program (SPSS version 19.0, SPSS Ibérica). Descriptive statistics (mean ± SD) were used for the groups. A one-way analysis of variance (ANOVA) followed by the Bonferroni test was used to compare the migration between groups. An unpaired t test was used to compare differences between pregnant and non-pregnant animals within the groups. Correlation between the volume of MB injected and the number of stained vertebral segments was evaluated using linear regression analysis and linear regression analysis forced through zero for pregnant and non-pregnant sheep. A *P*<0.05 was considered significant.

## Results

There were no significant differences in age, gestational age and body weight between the groups. Time of anaesthesia of all groups was 30±0.6 min and there were no significant differences on cardiopulmonary parameters during the entire study period in all animals, pregnant and non-pregnant. One of the pregnant ewes in group 2 was treated with a single bolus of 5 mL/kg of lactated Ringer for 10 minutes due to hypotension (Mean Arterial Pressure = 52 mmHg) after 20 minutes of anaesthesia.

Hanging-drop technique to ensure the correct needle placement was successful in all animals, pregnant and non-pregnant sheep.

The length of epidural cranial migration and number of stained vertebral body segments for non-pregnant and pregnant sheep are shown in Table 1. The mean number of vertebral body segments in group 3 (0.2 mL/kg), for both pregnant and non-pregnant animals, was significantly greater compared to groups 1 and 2 (0.05 and 0.1 mL/kg) respectively (*P*<0.001). Likewise, the mean number of vertebral body segments in group 2 was significantly greater compared to group 1 (*P*<0.001). Also, the cranial migration of the dye was significantly greater in group 3 compared to groups 1 and 2 (*P*<0.001), and when group 2 was compared with group 1 (*P*<0.001). Furthermore, significant differences were observed within the same groups between pregnant and non-pregnant sheep (*P*<0.01 for groups 1, 2, and 3).

**Table 1:** Mean ± standard deviation (SD) of body weight, length of epidural cranial migration and number of vertebral body segments dyed cranially by MB after epidural injection in anaesthetized non-pregnant and pregnant sheep.

	Non-pregnant			Pregnant		
MB volume (mL/kg)	0.05	0.1	0.2	0.05	0.1	0.2
Body weight (kg)	46.72±1.1	51.14±5.9	48.16±5.6	46.50±4.6	46.74±2.4	50.17±1.4
Length of epidural cranial migration (cm)	14.3±1.3^a^	22.4±6.5^a^ ^b^	37.16±3.8^a^ ^b^ ^c^	19.2±3.0^a^ ^b^ ^c^ ^d^	27.3±4.5^a^ ^b^ ^c^ ^d^ ^e^	41.6±1.8^a^ ^b^ ^c^ ^d^ ^e^
Number of stained vertebral body segments (cranial)	1.9±0.7^a^	4.9±0.9^a^ ^b^	7.7±0.6^a^ ^b^ ^c^	4.6±0.5^a^ ^b^ ^c^ ^d^	7.2±0.8^a^ ^b^ ^c^ ^d^ ^e^	12.5±0.6^a^ ^b^ ^c^ ^d^ ^e^
Range	L6–L5	L4–L3	L1–T13	L4–L3	L1–T13	T8–T9

a,b,c,d,e Same letters within a row indicate a statistical difference (*P*<0.001).

**Figure 1:**
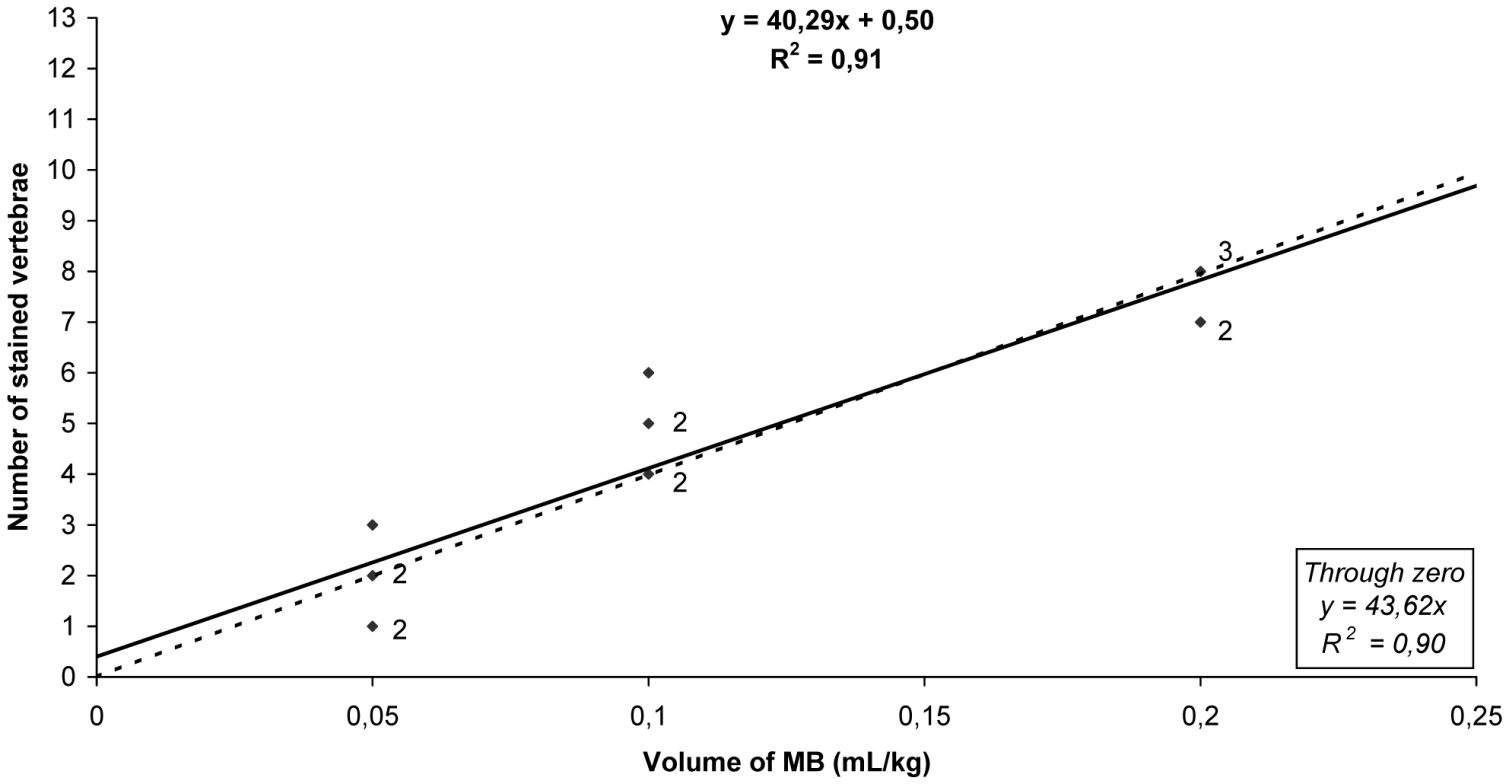
Regression analysis of the number of MB-stained vertebral body segments in non-pregnant sheep. Solid diamonds indicate individual data points, the solid line represents regression through the data, and the dotted line represents regression through zero. Small numbers adjacent to the data points indicate the number of data points represented by a single symbol.

**Figure 2:**
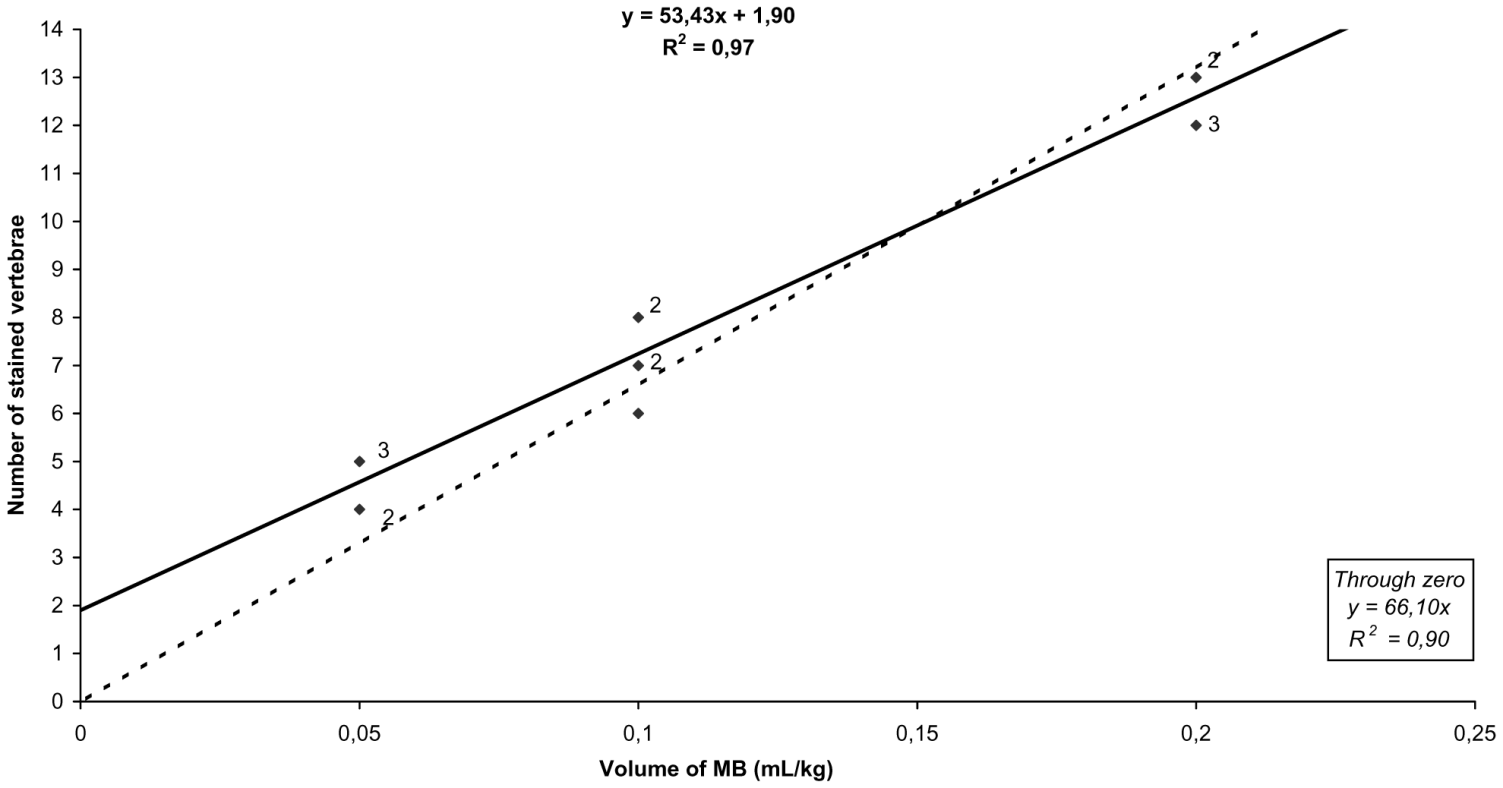
Regression analysis of the number of MB-stained vertebral body segments in pregnant ewes. Solid diamonds indicate individual data points, the solid line represents regression through the data, and the dotted line represents regression through zero. Small numbers adjacent to the data points indicate the number of data points represented by a single symbol.

There was a significant correlation between the injected volume and the number of vertebral body segments in both non-pregnant and pregnant sheep (coefficient of determination, *R^2^* = 0.91 and *R^2^* = 0.97; and forced through zero, *R^2^* = 0.90 and *R^2^* = 0.90, respectively) (Figure 1 and 2). Migration of the dye caudal to the lumbosacral space was observed in all animals, and 2.6±0.8 vertebral segments were stained in all groups.

## Discussion

The aim of studies that evaluate the distribution of MB injected into the epidural space is to identify a relationship between injection volume and the degree of cranial migration. There are several factors, described in the introduction, that influence the spread of drug solutions in the epidural space. To minimize such variables in the present study, we used animals with a similar age, gestational age, and body weight; and all injections were performed by the same investigator. The time from epidural injection to euthanasia (30 minutes) was considered sufficient for the migration of the solution, as analgesia for laparotomy in small ruminants was observed within 30 minutes after lumbosacral epidural injection of bupivacaine [18].

Our results show that the volume of MB injected into the epidural space in non-pregnant and pregnant sheep correlated directly with its cranial migration. The coefficient of determination (*R^2^* = 0.91 or *R^2^* = 0.97; and forced through zero, *R^2^* = 0.90 or *R^2^* = 0.90, in non-pregnant and pregnant sheep respectively) demonstrates the linearity and closeness of our data, showing that administration of 0.2 mL/kg in non-pregnant sheep and 0.1 mL/kg in pregnant ewes of MB in the lumbosacral junction (L7–S1) stained up to the T13–L1 vertebral body segment.

Based on the distribution of MB observed in our study, the administration of a local anaesthetic at these volumes in non-pregnant and pregnant sheep should provide useful analgesia in regions supplied by dermatomes dorso-caudal to the ribs and lumbar area. However, the correlation between the spread of MB and drug effect after the administration of a local anaesthetic into the epidural space has not been demonstrated [16], [19]–[21]. In these studies there was a wider distribution of MB in the epidural space than indicated by dermatomal analgesia. Nevertheless, in sheep the maximum degree of dermatome block obtained after the epidural injection of a volume of 0.16 mL/kg was T12–T13 [22], and this is a wider distribution than the number of stained vertebra observed after the epidural injection of 0.2 mL/kg MB in non-pregnant sheep [15]. This discrepancy is likely due to differences between species, although it could also be due to different local anaesthetics with different physicochemical properties, volumes and concentrations used in each study. For this reason, further studies are needed to determine the specific effect of the different volumes of local anaesthetic administered epidurally in sheep. However, the results of the current study could form the basis for determining and evaluating the pharmacodynamics of anaesthetics used in caudal epidural anaesthesia in pregnant ewes.

In the present study, sternal recumbency position of the ewes was used in order to ensure that the needle was inserted in the mid-line. Also, the relationship between body position and cranial migration of MB injected into the epidural space of canine cadavers has been investigated, and showed that the position and duration of recumbency time influence the cranial migration. A significantly greater cranial migration in the quadrant of the lateral recumbency was observed in dogs placed for 40 minutes [9]. To avoid a greater spread of the dye in a single lateral quadrant of the epidural space, the sheep remained in the same position, sternal recumbency, throughout the experiment.

However, caesarean section is one of the most common abdominal surgeries in small ruminants. The surgical approach with the animal in right lateral recumbency is often performed, and paravertebral and local infiltration at the incision site are used in sheep to produce regional anaesthesia [23]. Likewise, a lumbosacral epidural injection performed with the ewe in sternal recumbency and placed for 5 minutes in left lateral recumbency was useful in caesarean section of ewes associated with vaginal prolapse, vaginal and rectal prolapse, and dystocias [24], [25]. In our study we have not demonstrated the cranial migration of MB in lateral recumbency, so additional studies are required to determine if there are differences in the cranial migration between the two positions in pregnant and non-pregnant sheep.

In our study there were significant differences (*P*<0.01) between non-pregnant and pregnant animals within the same group regarding the cranial migration of MB. Moreover, the mean number of stained vertebral body segments in pregnant ewes of groups 1 and 2 was similar to groups 2 and 3 of non-pregnant sheep. The results observed in non-pregnant sheep are comparable to those described in anaesthetized Ripollesa non-pregnant sheep under similar conditions [15]. In that study, sheep were anaesthetized during 30 minutes, were positioned throughout the entire study period in sternal recumbency and no hypotension was observed. These results indicate that the volume administered epidurally has a greater cranial migration in pregnant animals than in non-pregnant ones, and a reduction in the volume by half should be done in pregnant ewes when compared to non-pregnant ones if a lumbosacral epidural anaesthesia is performed.

The differences in the cranial spread of MB between pregnant and non-pregnant sheep could be attributed to a number of factors. First, different studies have shown that the size of the epidural space is reduced in pregnant versus non-pregnant females [12], [13], [26]–[29]. The narrow epidural space could be due to the engorgement of the epidural veins; in humans the blood vessels are engorged in the first and third trimester of gestation [27], [29], secondary to increased systemic blood volume associated with pregnancy [27], [30], and by the direct compression on the inferior vena cava [26], [31]. Also, the engorgement could be due to the increase in water content in the connective tissue of the epidural space and the increase in the density of the vascular networks [27]. Further, in ruminants the abdominal pressure increases during inspiration because of displacement of the abdominal contents by the rumen, and since there are anastomosis between the veins of the thoracoabdominal cavity and the epidural veins [32], blood is forced into the epidural veins, increasing their size. This engorgement of the veins could be greater in pregnant animals than in non-pregnant, since the abdominal pressure may be higher in pregnant ones, due to the increased volume of the uterus in the latter.

Furthermore, in humans the effect of the pregnant uterus on the epidural venous plexus in different postures has been investigated, showing that in the ventral and supine position the pregnant uterus obstructs the vena cava, disturbing the return of the venous blood and causing engorgement of the venous plexus, whereas in lateral recumbency these changes were not observed [26], [29], [31]. The ewes in our study were in sternal recumbency, and the compression of the inferior vena cava by the enlarged uterus could be major in pregnant ewes and effect the spread of solutions injected into the epidural space. All these changes in the epidural space could facilitate the spread of local anaesthetics within the epidural space in pregnant animals.

Although in this study the body condition of the sheep were not evaluated, another possible explanation for the differences between pregnant and non-pregnant sheep is that in pregnancy the body condition score changes during the second trimester of gestation and fat accumulation is observed in pregnant sheep [33]. In this study, the net mobilization of lipids from subcutaneous adipocytes began around 100 days of gestation, and a rapid decrease was observed until day 105. However, due to the increase in energy intake during the last month of pregnancy, fat mobilization was retarded between 105 and 135 days of gestation. After day 135, net mobilization of lipids with fall in adipocyte mean volume occurred again. This fat accumulation could influence the epidural migration of drugs, resulting in a greater cranial migration in the pregnant ewes in our study compared to non-pregnant sheep; as Lee *et al.* [9] have shown that the existence of abundant epidural fat in the lumbosacral region contributes to variations in the spread of MB injected into the epidural space.

Finally, to calculate the volume of epidural anaesthesia according to the total body weight of pregnant ewes, we should consider the weight of the fetus, placenta, and fetal fluids. A volume reduction should be applied accordingly, since the fetus, placenta, and fetal fluids in pregnant ewes during the last trimester of gestation account for 5.5–9% of the total body weight in ewes carrying a singleton fetus and 9–13% of those carrying twins [34]. In our study, the volume in mL/kg of MB was calculated using the total body weight, as in clinical situations; there was no reduction in the volume administered to account for the pregnancy. This could have influenced the results, indicating increased cranial migration of MB compared to non-pregnant sheep. For this reason, it is recommended for precise epidural drug administration, specially during the last third of gestation, to take into account the weight of the fetus, placenta, and fetal fluids within the total body weight of pregnant ewes.

One last consideration is that the ewes in our study were in the last third of gestation (121.4±1.75 days) but were not at term. In ewes at term pregnancy, the cranial spread of MB injected into the epidural space could be slightly greater than that observed in this study, since the total weight of the fetus, placenta and fetal fluids should be slightly higher, and the engorgement of the epidural veins should also be slightly larger.

In conclusion, our results indicate that a volume of 0.2 or 0.1 mL/kg, injected into the lumbosacral epidural space of non-pregnant or pregnant sheep respectively, stains up the first lumbar vertebral body segment. These results should be a useful baseline for future investigations of epidural drug administration in pregnant animals. Furthermore, due to the differences between pregnant and non-pregnant sheep, the volume used in pregnant ewes should be reduced below that recommended for use in non-pregnant animals.
